# Isolation of Porcine Reproductive and Respiratory Syndrome Virus GP5-Specific, Neutralizing Monoclonal Antibodies From Hyperimmune Sows

**DOI:** 10.3389/fimmu.2021.638493

**Published:** 2021-02-22

**Authors:** Jordan E. Young, Cheryl M. T. Dvorak, Simon P. Graham, Michael P. Murtaugh

**Affiliations:** ^1^ College of Veterinary Medicine, University of Minnesota, St. Paul, MN, United States; ^2^ The Pirbright Institute, Pirbright, United Kingdom

**Keywords:** porcine reproductive and respiratory syndrome virus, humoral immunity, broad neutralization, B cell, monoclonal antibody, glycoprotein-5

## Abstract

Porcine reproductive and respiratory syndrome (PRRS) is a devastating disease which impacts the pig industry worldwide. The disease is caused by PRRS viruses (PRRSV-1 and -2) which leads to abortions and other forms of reproductive failure in sows and severe respiratory disease in growing pigs. Current PRRSV vaccines provide limited protection; only providing complete protection against closely related strains. The development of improved PRRSV vaccines would benefit from an increased understanding of epitopes relevant to protection, including those recognized by antibodies which possess the ability to neutralize distantly related strains. In this work, a reverse vaccinology approach was taken; starting first with pigs known to have a broadly neutralizing antibody response and then investigating the responsible B cells/antibodies through the isolation of PRRSV neutralizing monoclonal antibodies (mAbs). PBMCs were harvested from pigs sequentially exposed to a modified-live PRRSV-2 vaccine as well as divergent PRRSV-2 field isolates. Memory B cells were immortalized and a total of 5 PRRSV-specific B-cell populations were isolated. All identified PRRSV-specific antibodies were found to be broadly binding to all PRRSV-2 isolates tested, but not PRRSV-1 isolates. Antibodies against GP5 protein, commonly thought to possess a dominant PRRSV neutralizing epitope, were found to be highly abundant, as four out of five B cells populations were GP5 specific. One of the GP5-specific mAbs was shown to be neutralizing but this was only observed against homologous and not heterologous PRRSV strains. Further investigation of these antibodies, and others, may lead to the elucidation of conserved neutralizing epitopes that can be exploited for improved vaccine design and lays the groundwork for the study of broadly neutralizing antibodies against other porcine pathogens.

## Introduction

Porcine reproductive and respiratory syndrome virus (PRRSV) can spread rapidly among pigs through multiple routes of transmission, including aerosolization, causing severe respiratory disease that often leads to secondary infections and death in nursery age pigs (1). In sows, it is common to observe sudden loss of litters late in gestation, along with stillbirth and weak piglets in farrowed litters (1). PRRSV infection can also remain persistent in pigs for several months before it is completely cleared (2, 3). Because of these clinical manifestations and viral persistence, PRRSV remains an economically devastating disease in swine herds worldwide (4, 5).

Several antibody epitopes are suspected to be involved in neutralization of PRRSV, but there is a large gap in knowledge of how individual antibodies are capable of neutralizing PRRSV (6, 7). The majority of historical neutralization studies have concentrated on the PRRSV structural protein GP5, with interest in epitope B (amino acids 36-52), which has been proposed as an epitope for broad neutralization (8–12). It is important to note that while GP5 is required for the formation of viral particles, it is not required for virus to be infectious, suggesting inhibition of the GP5 viral protein alone may not lead to full neutralization of the virus (13). Along similar lines, several studies have also determined that GP5 specific antibody responses are non-neutralizing, however these studies appear to conflict with previously published studies (14–20). Recent work has determined that the PRRSV GP2-GP3-GP4 minor glycoprotein complex is key to viral entry into the host macrophage, through its interactions with CD163 (21–23). Based on these findings, other studies have also suggested sites of neutralization on GP4 and GP3 inducing renewed interest in the GP2-GP3-GP4 complex as a target for neutralization (24–28).

Passive immunization of sows with PRRSV-neutralizing antibodies have been shown to prevent viremia and viral transfer to the fetus, but an individual pig’s clearance of virus does not always correlate to a neutralizing antibody response (29, 30). The humoral immune response against PRRSV generates a large array of PRRSV-specific antibodies, but these antibodies vary in neutralizing capacity from non-neutralizing, to homologously neutralizing, and, rarely, to broadly neutralizing (31, 32). Current vaccines against PRRSV provide limited protection, often restricted to closely related viruses (33). Cross-protection has been shown to be achievable in pigs, but it does not always occur and seems to require multiple heterologous PRRSV exposures, such as vaccination as well as heterologous viral challenge (31, 34–36).

We theorize that exposure to diverse PRRSV strains enhances and improves the likelihood of the generation of broadly neutralizing antibodies. Therefore, to better evaluate PRRSV neutralizing antibodies, CD21^+^, IgG^+^ memory B cells from pigs whose sera broadly neutralized PRRSV were isolated and immortalized (37). Clonal populations of immortalized B cells that secreted PRRSV-specific antibodies were obtained. Antibodies isolated from these populations were mainly found to be GP5-specific and broadly binding to PRRSV-2 strains. One antibody was determined to neutralize homologous but not heterologous PRRSV strains.

## Materials and Methods

### Cells, Viruses, and *In Vitro* Infection

Archived samples from two parity 1 sows that had received two PRRSV ATP vaccinations and one or two PRRSV-2 field strain inoculations over a period of several months were collected 90 days after the final live virus exposure. Samples used for this study were peripheral blood mononuclear cells (PBMCs), splenocytes and serum.

Evaluation of antibody specificity and neutralization capacity was determined using the following MARC-145 cell adapted PRRSV strains: ATP (PRRSV-2, GenBank accession: EF532801.1), VR2332 (PRRSV-2, GenBank accession: MT269876), MN184 (PRRSV-2, GenBank accession: MT269877), 1-7-4 (PRRSV-2, GenBank accession: MN175677), 1-4-4 (PRRSV-2, GenBank accession: MT269878), 1-3-4 (PRRSV-2, GenBank accession: MT269879), 1-26-2 (PRRSV-2, GenBank accession: KF724400), SDEU (PRRSV-1, GenBank accession: MN175678) and Lelystad virus (PRRSV-1, GenBank accession: M96262.2). For antigen-baiting of PRRSV-specific B cells, PRRSV-2 VR2332 was labeled with Alexa Fluor-647 (AF647) as previously described for dengue virus (38). A phylogenetic tree showing the relationship between these PRRSV strains was created using both whole genome and ORF5 sequences, aligned with ClustalW using Mega version X gene analysis software (39). MARC-145 cells were grown and infected with PRRSV as previously described (34).

### B Cell Immortalization, Culture, and Flow Cytometry

B cell immortalization was performed starting with PBMCs from pigs showing evidence of heterologous neutralization which were previously isolated and cryopreserved (40). PBMCs were thawed from liquid nitrogen storage and cultured, at 2 × 10^6^ PBMCs per well, for 24 h in 24 well tissue culture plates in IMDM media containing 10% FBS with the addition of 5 × 10^4^ irradiated CD40L expressing L cells (CD40L-L cells; AIMM Therapeutics, The Netherlands), and 50 ng/ml recombinant murine IL-21 Fc fusion protein (IL-21; AIMM Therapeutics). PBMCs were then stained with fixable viability dye eFluor780 (1:200 dilution, eBioscience, San Diego, CA) to determine viability, PE mouse anti-porcine CD21 mAb (1:1,000 dilution, Abcam, Cambridge, MA), AF647 mouse anti-porcine IgG mAb (1:1,000 dilution, Cohesion Biosciences, London, UK) and biotinylated goat anti-porcine IgM polyclonal antibody (1:4,000 dilution, Bethyl Laboratories, Montgomery, TX) with Brilliant Violet 421 streptavidin to detect IgM labeling (1:3,000 dilution, BioLegend, San Diego, CA, USA). Cells (2-5 × 10^7^) were then sorted on a BD FACSAria II flow cytometer (BD Biosciences, San Jose, CA, USA) at the University of Minnesota Flow Cytometry Resource facilities and returned to a 24-well, tissue culture treated plate as described above, for 36 h. Sorted B cells were then mixed with the immortalization vector (GALV pseudotyped LZRS-Bcl6-2A-BclxL-IRES-GFP; AIMM Therapeutics), in equal volumes, and treated as previously described (37). Immortalized B cells were cultured for 7 to 9 days in IMDM media containing 10% FBS and evaluated for GFP expression, a marker of successful immortalization, *via* flow cytometry. Cells were then resorted *via* flow cytometry into populations of 20 B cells based on either binding to AF647 labeled PRRSV-2^+^/GFP^+^ (BNW7) or IgG^+^/GFP^+^ (BNW4) to 96-well, 300 µl volume, tissue culture treated plates, and returned to culture for 3 weeks. Cultured multi-cell populations were supplemented every 3 to 4 days with 25 µl of 10% FBS IMDM containing 2 µl of 5 µg/ml IL-21 and 20,000 irradiated CD40L-L cells. Multi-cell populations were examined for PRRSV specific antibodies by immunofluorescence and positive populations were resorted into single cell populations using flow cytometry and grown in culture for 3 to 4 weeks. Again, cultures were supplemented every 3 to 4 days with 25 µl of 10% FBS IMDM containing 2 µl of 5µg/ml IL-21 and 20,000 CD40L-L cells. Flow cytometry data was analyzed using FlowJo software v10 (Becton Dickinson, Franklin Lakes, NJ). CD40L expressing mouse L-cells, recombinant human IL-21, and the immortalization vector, GALV pseudotyped LZRS-Bcl6-2A-BclxL-IRES-GFP were graciously provided by AIMM Therapeutics (41). The retroviral vector containing Bcl-6 and Bcl-xL was generated by a for-profit company, AIMM Therapeutics, which makes the deep-frozen virus supernatant available. Obtaining the deep-frozen virus supernatant requires an MTA (http://www.aimmtherapeutics.com/partnering/academic-collaboration/) that includes financial obligations.

### Immunofluorescence Assay

An immunofluorescence assay (IFA) was performed to identify PRRSV-specific antibodies secreted from immortalized B cell populations. B cell supernatants from multi-cell populations were screened after 3 weeks of culture for the presence of virus binding antibodies by IFA. Supernatants from clonal populations were obtained after 5–7 days of culture and the presence of PRRSV-specific antibodies were evaluated by IFA. For the IFA assay, MARC-145 cells were plated at 1 × 10^4^ cells/well in 96-well plates in complete MEM media (cMEM; MEM with non-essential amino acids, 0.075% sodium bicarbonate, 25 mg/ml gentamycin sulfate, 10mM HEPES and 10% FBS). After two days in culture, 50 μl of 2 × 10^3^ TCID_50_/ml PRRSV strain VR2332 or ATP (MOI=0.1) was added to each well and incubated at 37°C, 5% CO_2_ for 1 h. Without removing the viral inocula, 150 μl of cMEM media was added to each well and cells were incubated for an additional 23 h. Media was then removed and cells were fixed using methanol. B cell supernatants (100 µl) or serum (100 µl of a 1:2000 dilution) were then added to fixed wells containing infected cells and incubated at 37°C, 5% CO_2_ for 1 h. Wells were washed and 50 µl of a 1:100 dilution of secondary antibody, FITC conjugated goat anti-pig IgG antibody (Bethyl Laboratories, Montgomery, TX), was added and incubated for 1 h. Wells were washed and cell nuclei were stained using bisbenzimide for 20–30 min. Stained monolayers were visualized using a Nikon TE2000 fluorescent microscope (Nikon, Tokyo, Japan) at the University of Minnesota CBS Imaging Center using SPOT basic imaging software (SPOT Imaging Solutions, Sterling Heights, MI, USA). A commercial PRRSV antibody, SR30-A at a 1:1000 dilution (RTI, Brookings, SD, USA) was used as a positive control. Serum from a PRRSV naïve pig was used as a negative control. At least two replicates were run for each antibody containing sample.

### Antigen-Antibody Binding Assay

Supernatants from B cell cultures and pig serum were examined for their ability to bind recombinant structural and non-structural PRRSV proteins using ELISA. PRRSV structural proteins, GP2, GP3, GP4, GP5, GP5-M, M 5’ (N-terminal 32 aa), M 3’ (C-terminal 88 aa), and nucleocapsid (N), and PRRSV non-structural proteins, NSP2P, NSP4, NSP7, NSP8, NSP11, that were previously expressed and purified, were examined (42–44). All PRRSV proteins were previously expressed in *E. coli*, contain a his-tag, and were purified using metal affinity chromatography (44). Correct folding of protein constructs was not verified. Proteins were constructed from PRRSV-2 VR2332 sequence, with the exception of GP2, GP3 and GP4, which were constructed from PRRSV-2 SD95-21 (GenBank accession: KC469618.1), which has a 99.98% nucleotide identity and 100% amino acid homology within GP2, GP3 and GP4 to VR2332, and were kindly provided by Dr. Ying Fang (University of Illinois, Champaign, IL, USA). ELISAs were performed as previously described (42, 45). Briefly, 96 well, ELISA plates were coated with 100 to 500 ng of recombinant PRRSV protein at 4°C overnight, followed by the addition of primary antibody from 100 µl of immortalized B cell culture supernatants or 100 µl of 1:50 dilution of pig serum, a secondary antibody of horseradish peroxidase (HRP)-conjugated goat anti-pig IgG antibody at a 1:100,000 dilution (Bethyl Laboratories), and color was developed using a TMB substrate solution (Seracare Life Sciences, Milford, MA, USA). Absorbance at 450nm (A_450_) was determined using a BioTek Epoch ELISA plate reader (BioTek, Winooski, VT, USA). Positive and negative controls were run on each plate, as described above. At least two replicates were run for each antibody containing sample.

### PRRSV Neutralization Assay

Neutralizing antibody assays were performed on MARC-145 cell monolayers as previously described (34). IgG-specific antibodies from B cell supernatants were isolated using the Thermo Scientific Pierce Protein A IgG Purification Kit (Thermo Fisher Scientific) following the manufacturer’s instructions. Antibodies were produced and purified at least 3 separate times. The concentration of IgG in each purified sample was determined using a pig IgG ELISA according to manufacturer’s protocols (Pig IgG ELISA Quantitation Set, Bethyl Laboratories). Isolated antibodies were adjusted to a concentration of 55ng/ul for use in the neutralization assay. Isolated antibodies (or control serum) were serial diluted 2-fold and mixed with the desired PRRSV strain. For both ATP and VR2332, infection was performed at an MOI of 0.06 and for SDEU the MOI was 0.02. The primary antibody SR-30A (mouse anti-PRRSV nucleocapsid antibody, SR30-A, RTI) was used at a 1:10,000 dilution and secondary antibody HRP-conjugated goat anti-mouse IgG (Bethyl Laboratories) was used at a 1:10,000 dilution. Color was developed using TMB solution (Seracare Life Sciences, Milford, MA, USA) and the optical density (OD) at 450 nm was obtained using a BioTek Epoch plate reader (BioTek). Background (uninfected MARC-145 cells) subtracted OD values were used to determine the percent inhibition of infection at each dilution as compared to virus only wells. At least 3 replicate neutralization assays were performed for each of the viruses using at least 2 batches of purified antibodies.

## Results

### Isolation, Immortalization, and Identification of PRRSV-Specific B Cells

**Figure 1:**
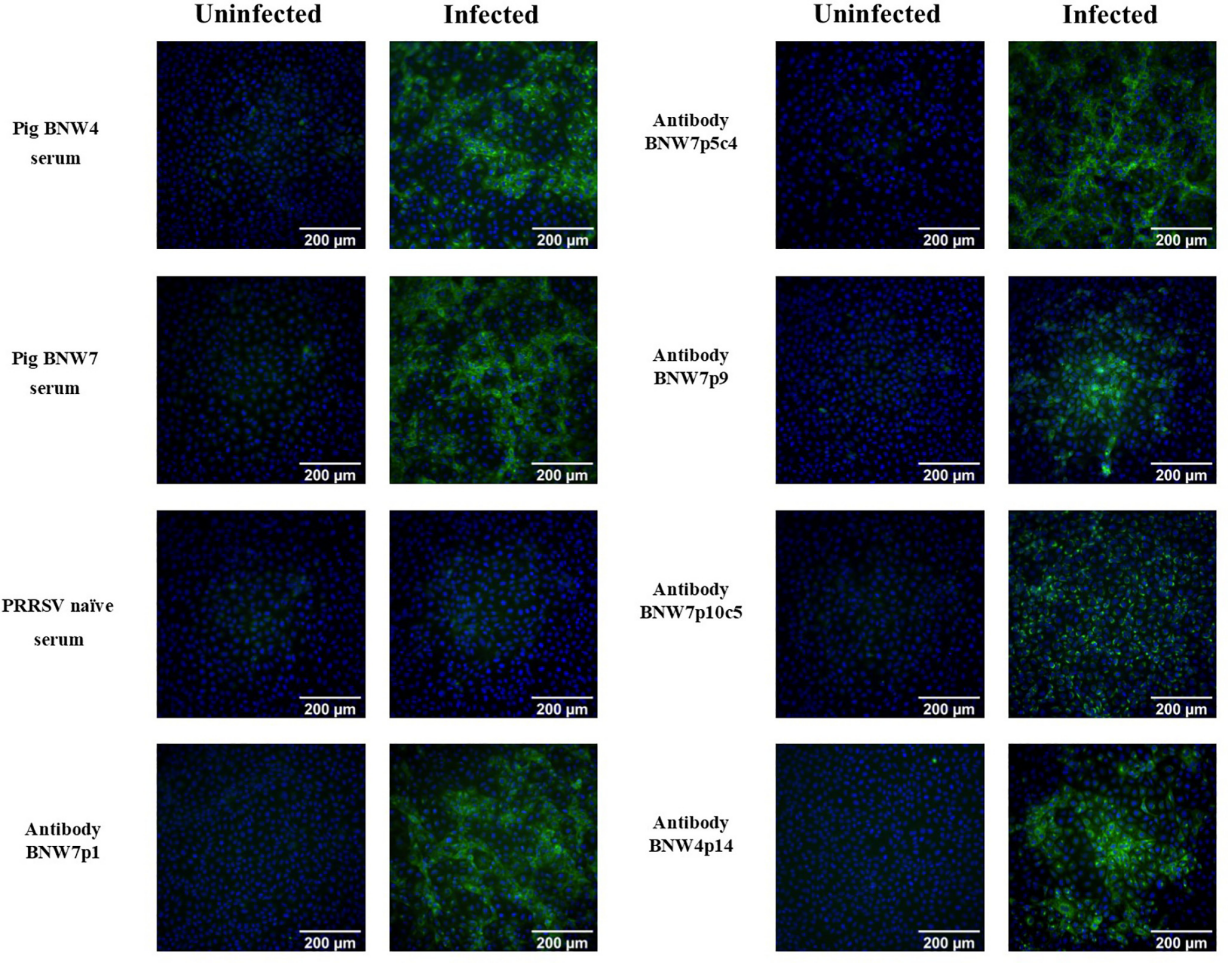
Immunofluorescence assay (IFA) visualization of antibody binding to porcine reproductive and respiratory syndrome (PRRSV) infected cells. Pig serum or antibody containing supernatants from immortalized B cell cultures were examined for their ability to bind PRRSV VR2332 infected MARC-145 cells and visualized using IFA. Uninfected MARC-145 cells are shown as a negative control of background fluorescence. Antibodies from BNW7p5c4 and BNW7p10c5 are from clonal B cell populations and antibodies from BNW7p1, BNW7p9, and BNW4p14 are from multi-cell B cell populations. Green indicates antibodies bound to PRRSV and Blue identifies nuclei stained with bisbenzimide. Shown are representative data from three repeat experiments.

Memory B cells from two pigs, BNW4 and BNW7, that had been exposed several times to both PRRSV ATP vaccine and field virus and whose sera neutralized a broad range of PRRSV strains (**Supplementary Figure 1**), were sorted and immortalized. A total of 480 multi-cell B cell populations were obtained from pig BNW7 through cell sorting based on binding of AF647 labeled PRRSV-2 VR2332, GFP presence (marker for immortalization), and surface expression of IgG. For pig BNW4, a total of 96 multi-cell B cell populations were sorted based on GFP and IgG expression. From 576 total screened populations, 5 PRRSV-specific immortalized B cell multi-cell populations were identified, four from pig BNW7 (p1, p5, p9 and p10) and one from pig BNW4 (p14). Based on the low rate of positive populations from both the BNW4 and BNW7 sorts, the virus-specific B cell enrichment was likely ineffective, and more than likely PRRSV specific B cells were identified by chance. Clonal populations were then isolated and propagated for two of the populations (BNW7p5c4 and BNW7p10c5). Supernatants from the 2 clonal populations and 3 multi-cell populations were examined for the ability of the secreted antibodies to bind VR2332 infected MARC-145 cells (**Figure 1**). A difference in the staining patterns between BNW7p10c5, which showed perinuclear staining, and the other antibodies, which showed cytoplasmic staining, was observed, suggesting that they are staining viral proteins that are localized to different areas of the cell (**Figure 1**). Two of the immortalized B cell clones, BNW7p5c4 and BNW7p10c5, were further characterized to identify expressed surface markers using flow cytometry and both were observed to express IgG and CD21, suggesting they were indeed memory B cells (**Supplementary Figure 2**).

### Antibody Binding to PRRSV Isolates

Antibody containing supernatants from immortalized B cells were evaluated for their ability to bind a panel of diverse PRRSV strains. A total of 7 PRRSV-2 (ATP, VR2332, MN184, 1-7-4, 1-4-4, 1-3-4, and 1-26-2) and 2 PRRSV-1 (SDEU and Lelystad virus) strains were used for examination of antibody binding (**Table 1**) and their phylogenetic relationships are shown in the supplementary material (**Supplementary Figure 3**). Serum from both BNW4 and BNW7 pigs were able to bind all the viruses examined (**Table 1**). Antibody containing supernatants from immortalized B cell cultures were able to bind all the PRRSV-2 strains examined, but neither of the PRRSV-1 strains examined (**Table 1**). Positive control antibody SR30-A was able to bind all viruses tested. Negative control serum from a PRRSV naïve pig was unable to bind any of the viruses tested.

**Table 1:** Antibody binding to porcine reproductive and respiratory syndrome (PRRSV) strains.

Virus sample	PRRSV-2							PRRSV-1	
	ATP	VR2332	MN184	1-7-4	1-4-4	1-3-4	1-26-2	SDEU	Lelystad
BNW4 serum	+	+	+	+	+	+	+	+	+
BNW7 serum	+	+	+	+	+	+	+	+	+
BNW4 p14	+	+	+	+	+	+	+	−	−
BNW7 p1	+	+	+	+	+	+	+	−	−
BNW7 p5c4	+	+	+	+	+	+	+	−	−
BNW7 p9	+	+	+	+	+	+	+	−	−
BNW7 p10c5	+	+	+	+	+	+	+	−	−

+, antibody containing sample bound to the PRRSV tested; −, no binding detected via IFA. Representative data from three repeat experiments.

### Antibody Binding to PRRSV Antigens

Antibodies secreted from immortalized B cells were evaluated for their ability to bind recombinant PRRSV-2 proteins (**Table 2**). Supernatants from immortalized multi-B cell populations BNW7p1, BNW7p9 and BNW4p14 and immortalized B cell clones BNW7p5c4, and BNW7p10c5 were tested by ELISA for reactivity against 13 recombinant PRRSV-2 proteins. Serum from pig BNW4 and BNW7 were used to evaluate the reactivity of all antibodies present within each animal. Serum from both animals showed strong reactivity to GP3, GP4, GP5, GP5-M complexed protein, M 5’ (N-terminal), N, NSP2P, NSP7, and NSP8 and weak reactivity to M 3’ (C-terminal) and NSP4 (**Table 2**). No binding of BNW7 serum was observed against, GP2 or NSP11, while BNW4 serum showed weak reactivity to both (**Table 2**). BNW7p9, BNW7p1, BNW7p5c4 and BNW4p14 bound to both GP5 and the GP5-M complex protein, suggesting these B cells secreted GP5 specific antibodies (**Table 2**). BNW7p10c5 did not bind any of the proteins examined, but it showed similar staining to that observed from antibodies specific to equine arteritis virus (EAV) nonstructural proteins, located in the endoplasmic reticulum (ER) of infected cells (46). Arterivirus non-structural proteins have been shown to concentrate in the host ER, where they are involved in control of host protein transcription and viral replication (46–48). This suggests that BNW7p10c5 may bind a viral non-structural protein, that we did not examine in this experiment.

**Table 2:** Antibody binding to porcine reproductive and respiratory syndrome (PRRSV) proteins.

Antibody	Structural proteins								Non-structural proteins				
	GP2	GP3	GP4	GP5	GP5-M	M 3’	M 5’	N	NSP2P	NSP4	NSP7	NSP8	NSP11
BNW4 Serum	+	+++	+++	+++	+++	+	+++	+++	++	+	+++	+++	+
BNW7 Serum	−	+++	+++	+++	+++	+	+++	+++	++	+	+++	++	−
BNW4 p14	−	−	−	+++	+++	−	−	−	−	−	−	−	−
BNW7 p1	−	−	−	++	+++	−	−	−	−	−	−	−	−
BNW7 p5c4	−	−	−	+++	+++	−	−	−	−	−	−	−	−
BNW7 p9	−	−	−	+	+	−	−	−	−	−	−	−	−
BNW7 p10c5	−	−	−	−	−	−	−	−	−	−	−	−	−

+++, high reactivity (OD value > 2); ++, moderate reactivity (OD value 1–2); +, low reactivity (OD value <1 and above the cut-off); – no reactivity (OD value below the cut-off value). Combined representative data from six repeat experiments.

### Viral Neutralization Activity of Antibodies Secreted by Immortalized B Cells

**Figure 2:**
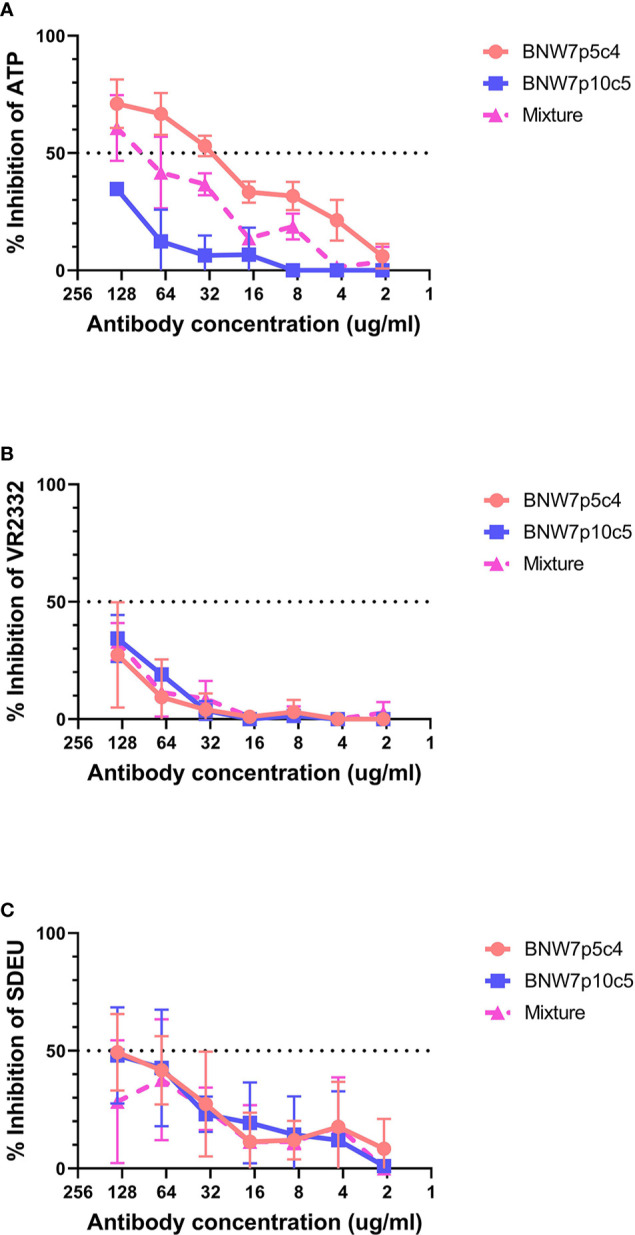
Neutralization activity of porcine mAbs against porcine reproductive and respiratory syndrome (PRRSV) strains. Antibodies secreted from clonal B cells BNW7 p5c4 and BNW7 p10c5 were purified from the supernatants using a protein A column. The IgG concentration was estimated using an IgG ELISA. A neutralization assay was performed against **(A)** PRRSV-2 ATP strain (homologous virus), **(B)** PRRSV-2 VR2332 (heterologous virus), and **(C)** PRRSV-1 SDEU (distant heterologous virus). To mimic a simplified polyclonal response the mixture sample contains an equal concentration of both BNW7p5c4 and BNW7p10c5 for a total IgG concentration as shown on the x-axis. Shown are representative data from three repeat experiments.

Antibodies from B cell clonal populations BNW7p5c4 and BNW7p10c5 were examined for their capacity to neutralize both homologous and heterologous PRRSV-2 strains. Because these pigs were vaccinated using ATP virus, the presence of antibodies against ATP was considered homologous neutralization. Heterologous neutralization was examined by looking at neutralizing activity against PRRSV-2 VR2332 strain, which the animals were not exposed to and which has a 10% difference in nucleotide sequence as compared to the ATP strain (**Supplementary Figure 3**). Serum antibodies from both pigs were able to neutralize ATP, VR2332, and SDEU viruses with extremely high neutralization titers against the PRRSV-2 strains (**Supplementary Figure 1**). Antibodies from supernatants were concentrated to ensure that lack of neutralizing activity was not due to low antibody levels and the concentration of IgG was determined in order to equalize antibody concentrations between clones. The highest antibody level tested was 137.5 µg/ml of IgG for BNW7p5c4 and BNW7p10c5. Each concentrated supernatant was examined for its neutralizing ability starting at 137.5 µg/ml and diluted 2-fold down to 2.1 µg/ml. A 50/50 mixture of antibodies from the two clones, BNW7p5c4 and BNW7p10c5, were tested for neutralizing activity to examine a possible polyclonal neutralization response. Concentrated supernatants from the GP5-binding BNW7p5c4 clone were observed to have a 50% neutralization titer against ATP virus at a concentration of 34.4 µg/ml IgG (**Figure 2A**). BNW7p10c5 was unable to neutralize ATP virus. The mixed sample containing both BNW7p5c4 and BNW7p10c5 was neutralizing, similar to what would be expected of a similar concentration of BNW7p5c4 supernatant alone (**Figure 2A**). This suggests that despite the ability of BNW7p10c5 to bind all PRRSV-2 viruses examined, it was non-neutralizing. Neither B cell supernatant (BNW7p5c4 or BNW7p10c5) was able to neutralize VR2332 or SDEU viruses, even when mixed together (**Figures 2B, C** **)**, suggesting the neutralizing activity of BNW7p5c4 was specific to the ATP strain even though it was able to bind all PRRSV-2 strains examined, as shown in **Table 1**.

## Discussion

The PRRSV envelope glycoprotein GP5 has long been thought to be the major antigen involved in virus neutralization (8, 11, 49, 50). GP5 is encoded by the ORF5 gene, which has been widely used for classification and analysis of PRRSV strains due to its immunological significance and polymorphic nature (51). Sequencing of PRRSV to examine relationships between strains has mainly been performed using ORF5 sequences and RFLP typing (51–53). Numerous neutralization studies have concentrated solely on the PRRSV GP5 protein and early in infection pigs show a strong antibody response to the GP5 protein (49, 50, 54). In this study we showed a similar propensity toward GP5 immunity with 4 out of our 5 PRRSV-binding immortalized B cell populations secreting GP5-specific antibodies, thus pointing toward GP5 being a dominant antigen in the anti-PRRSV immune response.

This study identified and isolated porcine derived PRRSV neutralizing antibodies and confirmed that GP5 contains a neutralizing epitope. Several studies have claimed that GP5 specific antibodies are non-neutralizing, however the data generated in this study contradicts these findings (19, 20). Porcine GP5-specific mAb BNW7p5c4 was shown to be able to achieve a 50% inhibition of homologous ATP virus at 34.4 µg/ml (**Figure 2A** **)**. Previous research determined that while GP5 is essential for virus formation and is responsible for adhesion to the host cell, it is not essential for virus entry (22, 55, 56). Li et al. showed that titers of antibody against the ectodomain of GP5 did not correlate with viral neutralizing antibody titers, suggesting that GP5 is not solely responsible for neutralization (20). It has also been demonstrated that pigs lacking sialoadhesin (Sn/Siglec-1/CD169) (the host macrophage protein that GP5 interacts with) are permissive to PRRSV infection, while pigs lacking CD163 (the host protein in which the GP2-GP3-GP4 complex interacts with) are completely resistant to virus infection (57, 58). Based on current knowledge of PRRSV infection and historically ineffective GP5-based vaccine platforms, full sterilizing immunity may not be achievable by induction of GP5 antibody alone (18, 20, 29, 59). More likely, the GP5-specific antibody, BNW7p5c4, inhibits infection but does not permanently prevent it. Our data, along with information from the literature, suggests that the BNW7p5c4 antibody is able to block viral binding to the host cell, thus lowering the ability of the virus to bind and infect the cell. However, since the GP2-GP3-GP4 complex on the virus remains intact and functional, rare viral entry events may still occur allowing for very low levels of infection. This theory on GP5 neutralization would explain some of the confusion and disagreement about the role of GP5 in neutralization. This could also present a reason why the virus persists, as the majority of neutralizing antibodies that we identified are directed toward GP5 and not the proteins responsible for viral entry (GP2, GP3, and GP4).

Homologous neutralization of virus is the most common outcome of exposure to PRRSV, either through vaccination or natural infection. In this study we identified that antibodies secreted from GP5-specific BNW7p5c4 B cells can neutralize homologous virus (ATP), but not heterologous virus (VR2332) (**Figure 2**). This outcome is not surprising, as antibodies against homologous virus are very common in pigs exposed to PRRSV, however the ability of the antibody to bind the VR2332 strain, but not neutralize it is an interesting observation. It appears the isolated mAb, BNW7p5c4, has different properties depending on which virus it is bound to; becoming a neutralizing antibody against ATP, but a non-neutralizing antibody against VR2332. The cause of this functional discrepancy is not known. It is possible that antibody avidity (total strength of antibody binding to antigen) to the epitope differs between the viruses, with stronger, long lasting binding to homologous virus and a short duration, weak binding to heterologous virus, which contributes to its neutralizing ability. Longer periods of antigen-antibody interactions should, in theory, improve elimination of a pathogen and increase positive feedback signals to B cells. Very few published studies have investigated antibody avidity to PRRSV. Ko et al. observed that vaccinated pigs that produced antibodies with high relative avidity indexes (RAI) had decreased and shortened viremia in response to viral challenge (60). Similar results were shown by Islam et al., where longer periods of viremia, as opposed to either quick clearance of virus or rebound infection, were associated with the development of heterologous and broadly neutralizing antibodies (61). It is possible that avidity also plays a role in the development of cross-neutralizing antibodies, with high avidity antibodies being more likely to cross-neutralize diverse viral strains.

The development of broadly neutralizing antibodies against PRRSV is rare and difficult to replicate (32). Based on our findings that all five isolated B cell populations secreted broadly binding antibodies against a diverse group of PRRSV-2 strains, we propose that these antibodies were generated *via* repeated positive antigen selection of a conserved region of GP5. Many previous studies have shown that multiple varied virus and vaccine exposures lead to improved immunity and most likely broad PRRSV binding, as observed in this study (31, 34, 36, 62).

This study shows the first ever isolation of a porcine PRRSV neutralizing mAb, it confirms that PRRSV GP5 contains a neutralizing epitope, and it suggests that broad binding, non-neutralizing antibodies may precede those that are broadly neutralizing. However, we propose, based on known viral mechanics and our results, that while GP5-specific antibodies aid in virus neutralization, they are most likely not the only PRRSV epitope-specific antibodies involved. In this study, we showed that pigs exposed to an array of genetically distant PRRSV strains over time, developed antibodies that were broadly reactive to diverse PRRSV. Future vaccines against PRRSV may be more effective at inducing protection against a broad range of PRRSV if they contain multiple isolates or genetically distinct epitopes that are present in the field. The addition of a multi-strain vaccine booster may also increase vaccine effectivity against a broad range of PRRSV strains. While these results shed new light on PRRSV neutralization, much is still unknown. The isolation of additional porcine-derived antibodies using the techniques used in this study should uncover further information about PRRSV neutralization. In particular, isolation of GP2, GP3, and GP4 specific antibodies will likely uncover additional neutralizing or potentially broadly neutralizing antibodies and combining monoclonal antibodies with binding specificities to different viral proteins may elucidate methods leading toward broadly neutralizing antibody activity.

## Data Availability Statement

The data sets presented in this study can be found in online repositories. The names of the repository/repositories and accession number(s) can be found below: https://www.ncbi.nlm.nih.gov/genbank/, EF532801.1, MT269876, MT269877, MN175677, MT269878, MT269879, KF724400, MN175678, M96262.2.

## Author Contributions

JY, CD, SG, and MM conceived the study and participated in its design and coordination. JY performed experiments and interpreted results. JY wrote the first draft of the manuscript. JY, CD, and SG contributed to manuscript revision and read and approved the final manuscript. All authors contributed to the article and approved the submitted version.

## Funding

Funding for this project was provided by USDA NIFA grant 2016-67015-24928 and JY was partially funded through the Sam Maheswaran Graduate Fellowship. SG is supported by a UKRI Biotechnology and Biological Sciences Research Council (BBSRC) Institute Strategic Programme Grant to the Pirbright Institute (BBS/E/I/00007031).

## Conflict of Interest

The authors declare that the research was conducted in the absence of any commercial or financial relationships that could be construed as a potential conflict of interest.
