# Supplementary material for: Isolation of Porcine Reproductive and Respiratory Syndrome Virus GP5-Specific, Neutralizing Monoclonal Antibodies From Hyperimmune Sows
Source: Front Immunol. 2021 Feb 22;12:638493. doi: 10.3389/fimmu.2021.638493 (PMC7937800; doi:10.3389/fimmu.2021.638493)
Supplement: Supplementary file 1 [file DataSheet_1.docx]

Supplementary Material

## Supplementary Figures


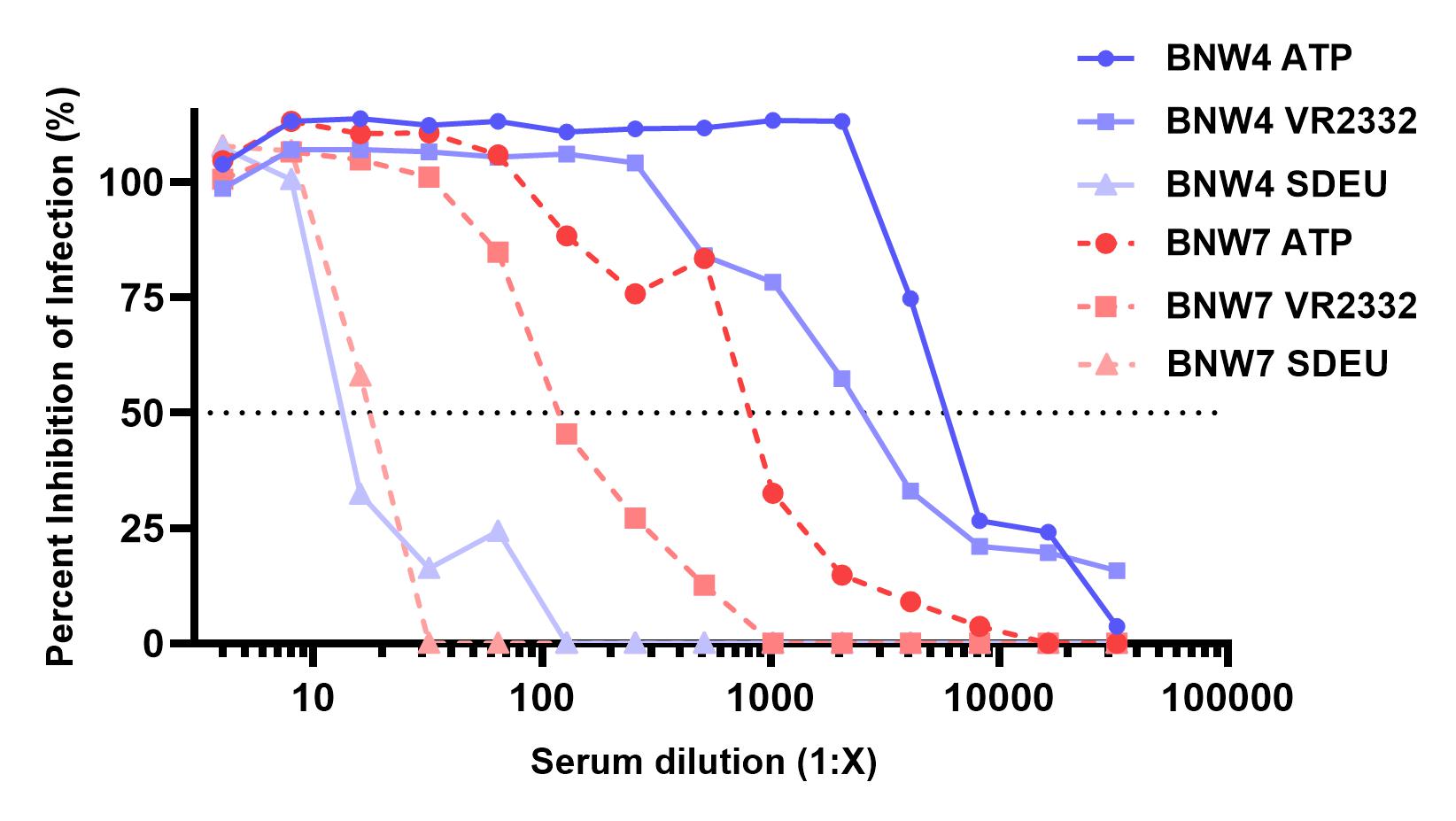


Supplementary Figure 1: Serum neutralization titers of diverse PRRSV isolates. The neutralizing titer of serum from pigs BNW4 and BNW7 was evaluated against the homologous PRRSV-2 ATP, heterologous PRRSV-2 VR2332, and heterologous PRRSV-1 SDEU isolates. The 50% neutralizing titer can be observed where the serum dilution crosses the dotted line at 50% inhibition.


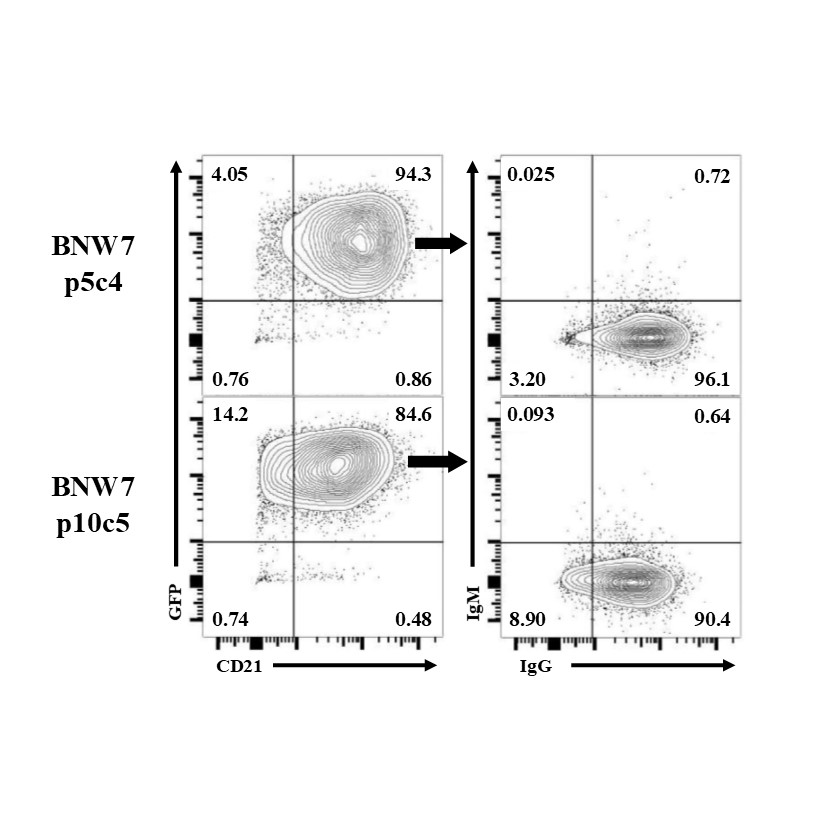


**Supplementary Figure 2:** Surface marker characterization of PRRSV-specific B cell clones BNW7p5c4 and BNW7p10c5. PRRSV-specific BNW7 clones p5c4 and p10c5 were evaluated for expressed cell surface markers via flow cytometry. Cells were initially gated on lymphocytes, single cells and live cells followed by intracellular GFP, an indicator of successful immortalization, and surface CD21 expression. Double positive GFP and CD21 cells were further characterized for IgM and IgG surface expression.


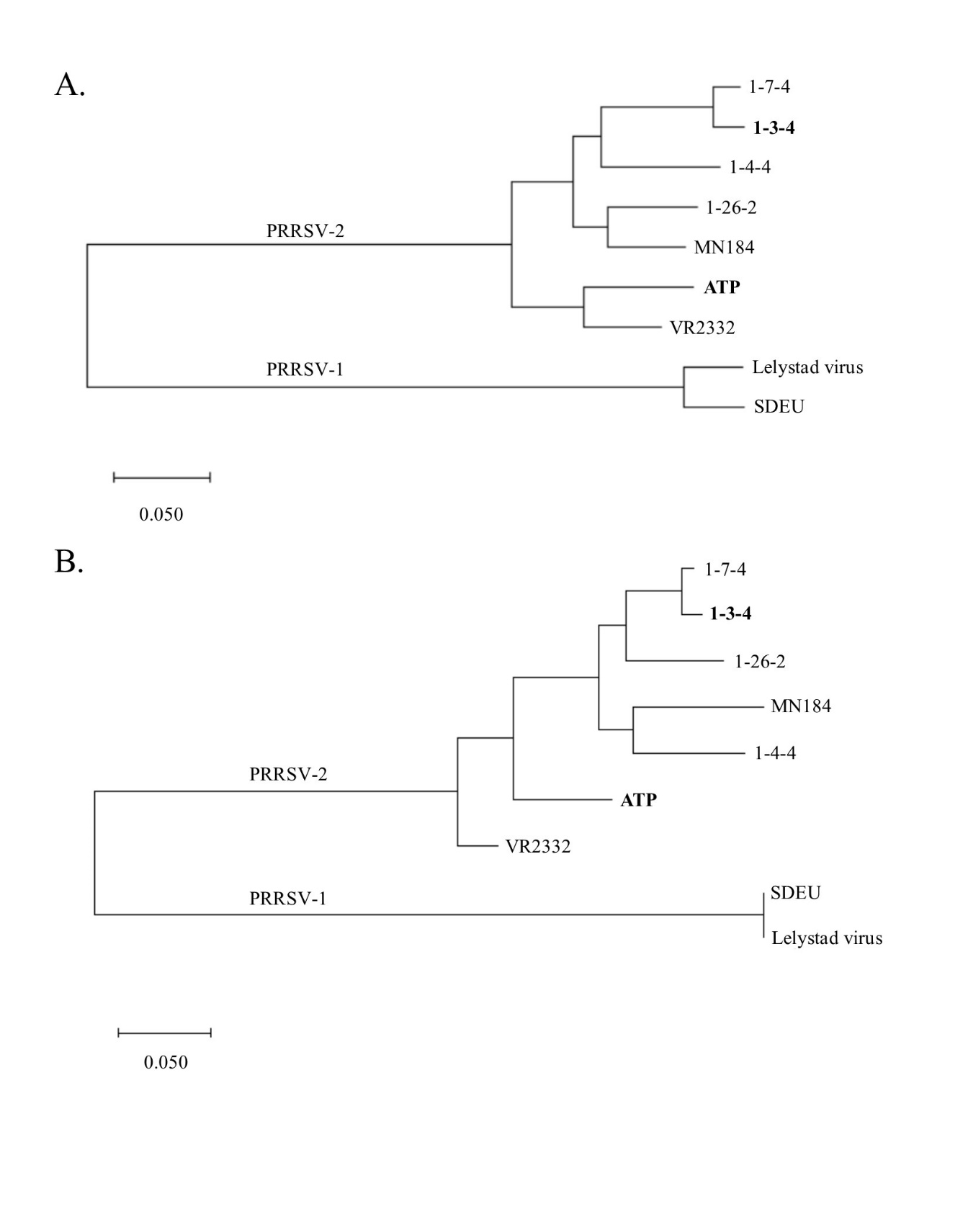


Supplementary Figure 3: Phylogenetic tree of PRRSV strains examined for antibody reactivity.

Whole genome sequences (A) and ORF5 sequences (B) were obtained and their genetic relatedness was examined using the neighbor-joining method within MEGA-X software [39]. The percent identity between the ATP virus and the other strains was determined identifying the percent identity against the whole genome and ORF5 sequence for 1-7-4 (82.8%, 87.5%), 1-3-4 (82.6%, 86.7%), 1-4-4 (82.7%, 87.1%), 1-26-2 (83.2%, 86.0%), MN184 (84.1%, 83.7%), VR2332 (90.3%, 89.6%), Lelystad virus (41.9%, 43.3%,), and SDEU (42.4%, 43.3%), respectively. All strains were used to examine the ability of antibodies secreted from immortalized B cells to bind diverse PRRSV strains. Strains in bold (ATP and 1-3-4) indicates the pigs examined had previous exposure to this virus through either vaccine or live inoculation.
